# Dietary, environmental, and genetic risk factors of Extensive Macular Atrophy with Pseudodrusen, a severe bilateral macular atrophy of middle-aged patients

**DOI:** 10.1038/s41598-018-25003-9

**Published:** 2018-05-01

**Authors:** Aymeric Douillard, Marie-Christine Picot, Cécile Delcourt, Sabine Defoort-Dhellemmes, Nour Al-Dain Marzouka, Annie Lacroux, Xavier Zanlonghi, Isabelle Drumare, Elsa Jozefowicz, Béatrice Bocquet, Corinne Baudoin, Sarah Perez-Roustit, Sophie Arsène, Valérie Gissot, François Devin, Carl Arndt, Benjamin Wolff, Martine Mauget-Faÿsse, Maddalena Quaranta, Thibault Mura, Dominique Deplanque, Hassiba Oubraham, Salomon Yves Cohen, Pierre Gastaud, Olivia Zambrowski, Catherine Creuzot-Garcher, Saddek Mohand Saïd, José-Alain Sahel, Eric Souied, Solange Milazzo, Rocio Blanco Garavito, Vasiliki Kalatzis, Bernard Puech, Christian Hamel, Isabelle Audo, Isabelle Meunier

**Affiliations:** 1 CHRU Montpellier, Clinical Investigation Center (CIC) & Clinical Research and Epidemiology Unit (URCE), Montpellier, France; 2 INSERM, CIC 1411 Montpellier, France; 3 University of Bordeaux, ISPED, F-33000 Bordeaux, France; 4 Inserm, U1219 - Bordeaux Population Health Research Center, F-33000 Bordeaux, France; 5 Service d’Exploration de la Vision et Neuro-ophtalmologie, Hôpital Robert Salengro, CHU de Lille, France; 6 Centre de Référence Maladies Sensorielles Génétiques, Hôpital Gui de Chauliac, University of Montpellier, Institute for Neurosciences of Montpellier INSERM U1051, Montpellier, France; 7 Eye Clinic Sourdille Jules Verne, Nantes, France; 8 University Lille, Inserm, CHU Lille, CIC 1403 – Centre d’investigation clinique, F-59000 Lille, France; 9 Eye Clinic, Hôpital de Tours, CHRU de Tours, Tours, France; 10 Inserm 1415, Centre d’investigation clinique, CHRU de Tours, Tours, France; 11 Eye Clinic, Centre Paradis, Monticelli, Marseille, France; 12 Eye Clinic, Hôpital Robert Debré, CHRU de Reims, France; 13 Eye Clinic, Maison Rouge, Strasbourg, France; 14 Fondation Adolphe de Rothschild, 25 rue Manin, 75019 Paris, France; 15 Centre Ophtalmologique Rabelais, Lyon, France; 16 Eye Clinic, Hôpital Intercommunal, Créteil, France; 17 Centre d’Imagerie Laser, Rue Antoine Bourdelle, Paris, France; 18 Eye Clinic, Hôpital Saint Roch, CHU de Nice, Nice, France; 19 Eye Clinic, Hôpital Universitaire de Dijon and Eye nutrition and signaling group, INRA, Dijon, France; 20 Sorbonne Université, UPMC Univ Paris 06, INSERM, CNRS, Institut de la Vision, 17 rue Moreau, 75012 Paris, France; 21 CHNO des Quinze-Vingts, DHU Sight Restore, INSERM-DHOS CIC1423, 28 rue de Charenton, 75012 Paris, France; 22 Institute of Ophthalmology, University College of London, London, EC1V 9EL UK; 23 Académie des Sciences, Institut de France, Paris, France; 24 Department of Ophtalmology, Amiens University Hospital, Paris, France

## Abstract

EMAP (Extensive Macular Atrophy with Pseudodrusen) is a maculopathy we recently described that shares pseudodrusen and geographic atrophy with Age-related Macular Disease (AMD). EMAP differs from AMD by an earlier age of onset (50-55 years) and a characteristic natural history comprising a night blindness followed by a severe visual loss. In a prospective case-control study, ten referral centers included 115 EMAP (70 women, 45 men) patients and 345 matched controls to appraise dietary, environmental, and genetic risk factors. The incidence of EMAP (mean 2.95/1.10^6^) was lower in Provence-Côte d’Azur with a Mediterranean diet (1.9/1.10^6^), and higher in regions with intensive farming or industrialized activities (5 to 20/1.10^6^). EMAP patients reported toxic exposure during professional activities (OR 2.29). The frequencies of common AMD complement factor risk alleles were comparable in EMAP. By contrast, only one EMAP patient had a rare AMD variant. This study suggests that EMAP could be a neurodegenerative disorder caused by lifelong toxic exposure and that it is associated with a chronic inflammation and abnormal complement pathway regulation. This leads to diffuse subretinal deposits with rod dysfunction and cone apoptosis around the age of 50 with characteristic extensive macular atrophy and paving stones in the far peripheral retina.

## Introduction

**Figure 1:**
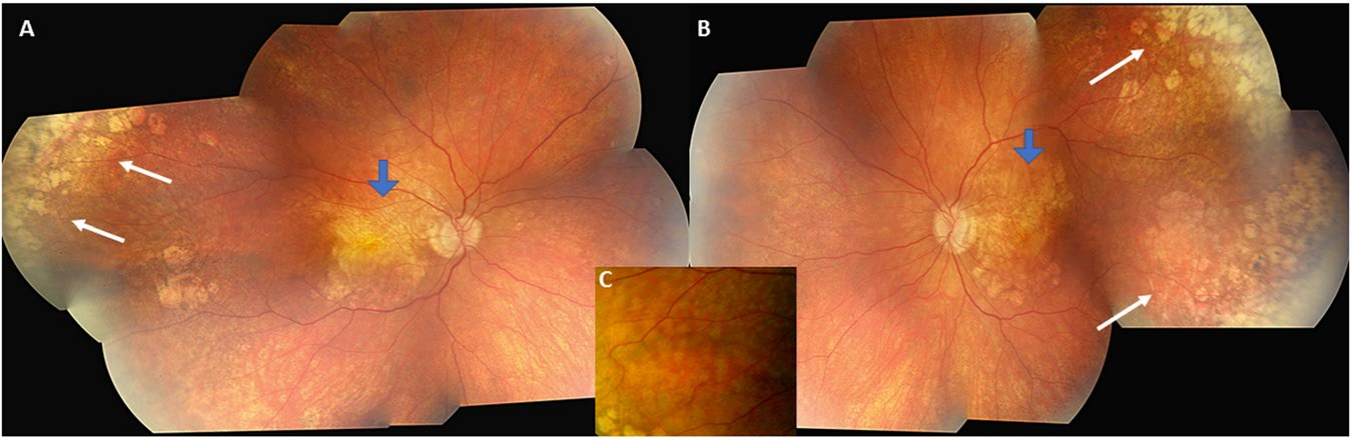
Fundus photographs of a 56-year-old woman with an EMAP disease. Visual acuity is 20/400 in both eyes. (**A**,**B**) There is a bilateral and symmetrical extensive geographic macular atrophy with a diameter larger in vertical than horizontal (beneath blue arrows) in both eyes (A right and B left-eye). Atrophic lesions are also noted in the far peripheral and temporal retina in both eyes (white arrows). The extension of the macular atrophy and the association with peripheral small patches of atrophy is not common in AMD. (**C**) Color-magnified photograph of the midperipheral retina that reveals the striking density of pseudodrusen.

**Figure 2:**
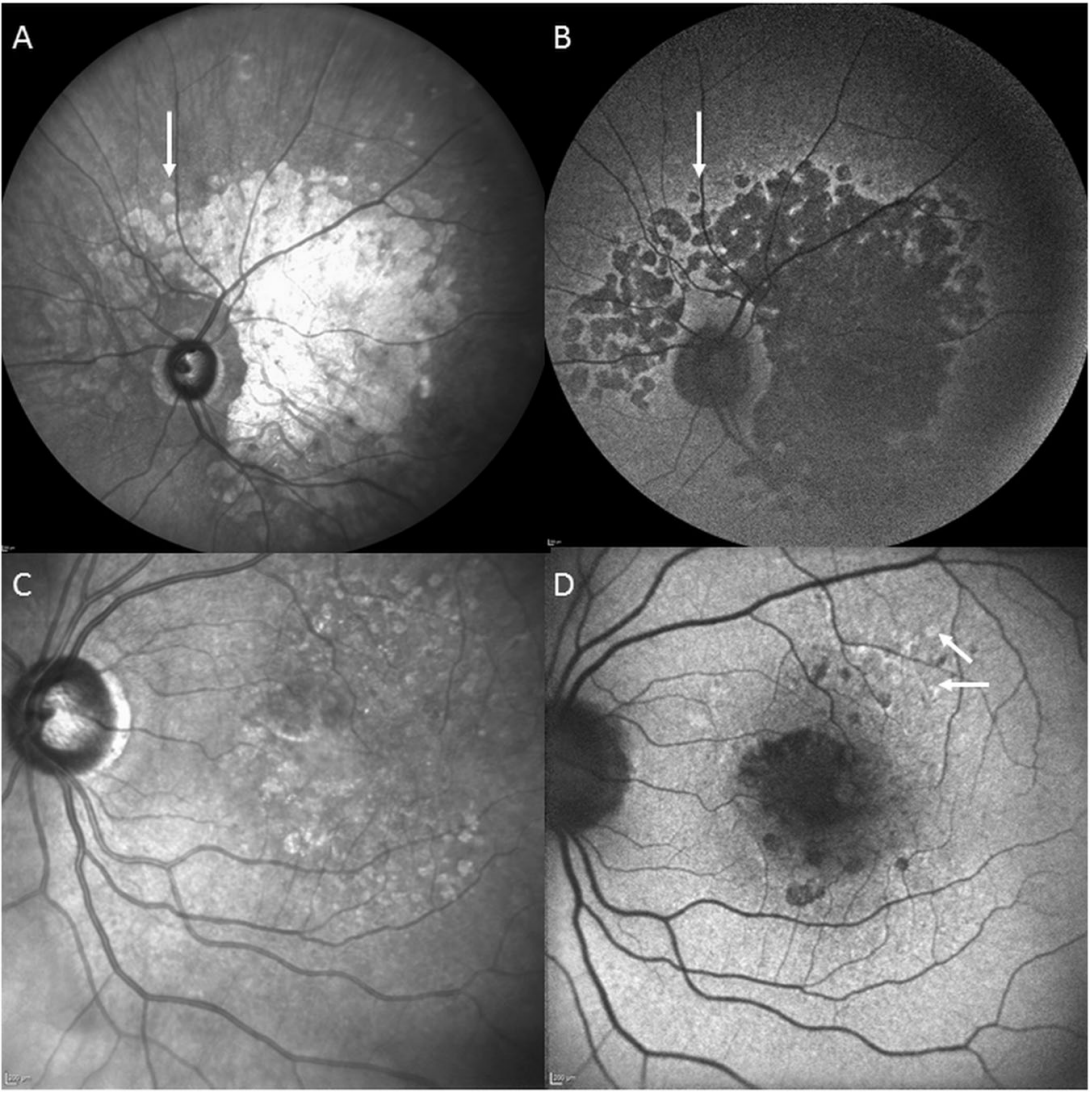
Extensive macular atrophy with pseudodrusen (EMAP) compared to atrophic age-related macular disease (AMD) with pseudodrusen. In EMAP, macular atrophy is extensive with a larger vertical axis passing over the optic nerve head (white arrow) to reach the nasal part of the peripapillary retina (**A**). The atrophy is dark, well-delineated on autofluorescence photograph (**B**). In AMD, there are several patches of macular atrophy restricted to the macular zone (**C**) surrounded by pseudodrusen and classic AMD drusen. On FAF, the atrophic patches are dark and surrounded by mild hyperautofluorescent AMD drusen (**D**, white arrows).

Extended Macular Atrophy with Pseudodrusen (EMAP) is a distinct macular disease characterized by a bilateral and symmetric extensive geographic atrophy involving the posterior pole without foveal sparing (Fig. 1)^1^. The macular atrophy is systematically associated with diffuse pseudodrusen lining the entire posterior pole and the midperiphery. Pseudodrusen (PSD) and macular atrophy are also encountered in age-related macular degeneration (AMD, Fig. 2), the most frequent macular disease occurring after the age of 60 years in high-incomes countries. In AMD, these lesions occur at late stages in the seventh to eighth decades of life^2–5^. By contrast, in EMAP these common elementary lesions are noted at the beginning of the disease in the fifth decade. Night blindness is a systematic inaugural clinical sign of EMAP, rarely reported in AMD. In addition, AMD is a perifoveopathy with a slow rate of progression and long term foveal preservation. By contrast, EMAP atrophy is strikingly rapidly progressing throughout the entire posterior pole with a larger vertical axis and a lack of foveal preservation. Furthermore, the majority of EMAP patients also present with paving stone lesions in the far peripheral retina (Fig. 1). EMAP also shares the pseudodrusen lesions and a fast progression of the atrophy with diffuse trickling geographic atrophy (DTGA), an early and severe form of AMD. But in contrast to EMAP, patients with DTGA frequently have severe vascular or cardiac disorders.

**Figure 3:**
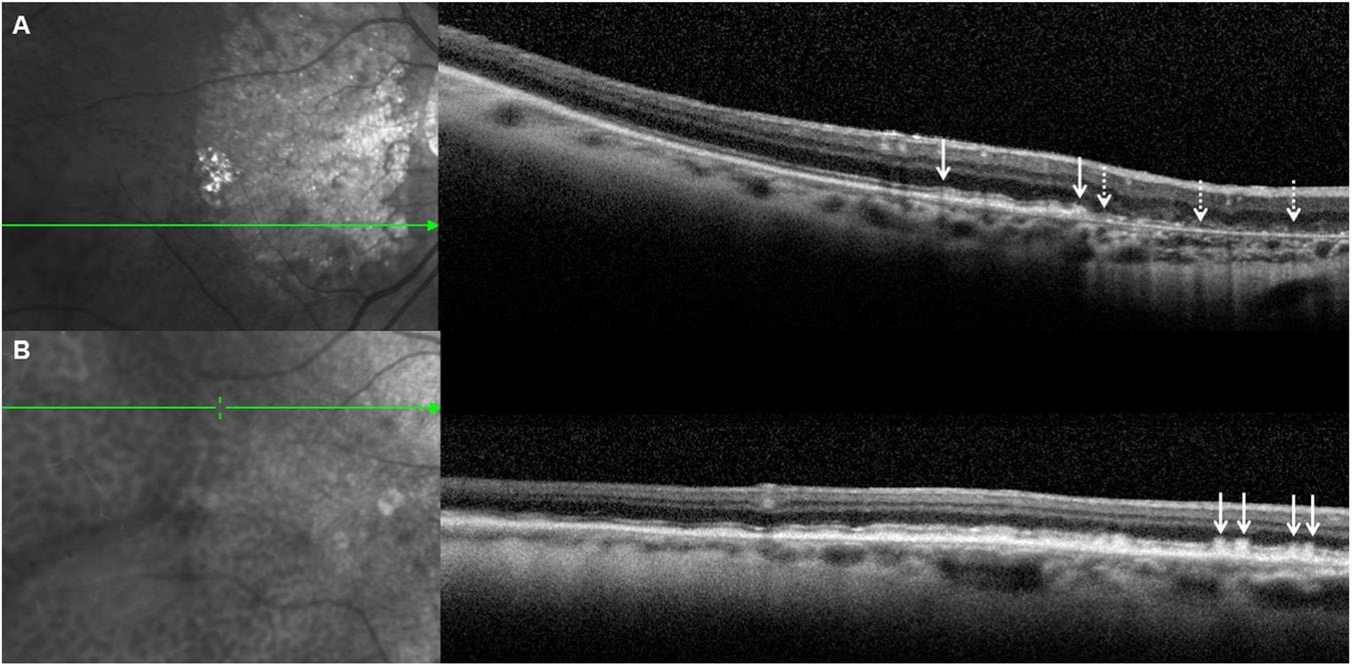
SD-OCT pattern in EMAP-pseudodrusen (**A**) and in age-related pseudodrusen (**B**). The deposits are above the RPE in both cases. In EMAP (**A**), the deposits have a diffuse pattern (between white arrows) adjacent to the macular atrophic lesion (dotted line arrows). In AMD (**B**), pseudodrusen have a nodular pattern (white arrows).

Pseudodrusen (PSD) are distinguished from other classic AMD drusen by their fundus appearance and their specific intraretinal localization (Fig. 2)^6–8^. PSD reproduce interlacing yellowish lesions enhanced by blue light. In EMAP, PSD are noted throughout the macular area and the whole peripheral retina, whereas in AMD, they are mainly restricted to the macular zone. On spectral domain optical coherence tomography (SD-OCT), PSD are not located beneath the retinal pigment epithelium (RPE) like classic AMD drusen, but in the subretinal space at the level of the RPE or between the RPE and the photoreceptors. Furthermore, in EMAP, PSD show a diffuse pattern whereas in AMD a dot pattern is more common (Fig. 3). PSD are noted in AMD but at a later stage, at a mean age of approximately 80 years in all multimodal imaging studies.

We undertook a large national clinical case-control study to identify EMAP risk factors and to compare them with those of late atrophic AMD with or without pseudodrusen. We previously highlighted an inflammatory mechanism underlying EMAP reflected by abnormal total hemolytic complement (CH50) and complement component 3 (C3) serum levels, and eosinophilia^9^. By contrast, systemic AMD risk factors such as blood pressure, body mass index, high-density lipoprotein (HDL) and cholesterol, were not detected^9^. This first study also excluded a role for medications with toxic retinal effects. Herein, we focus on dietary, environmental, and genetic risk factors associated with EMAP, as well as geographic distribution of cases.

## Results

**Figure 4:**
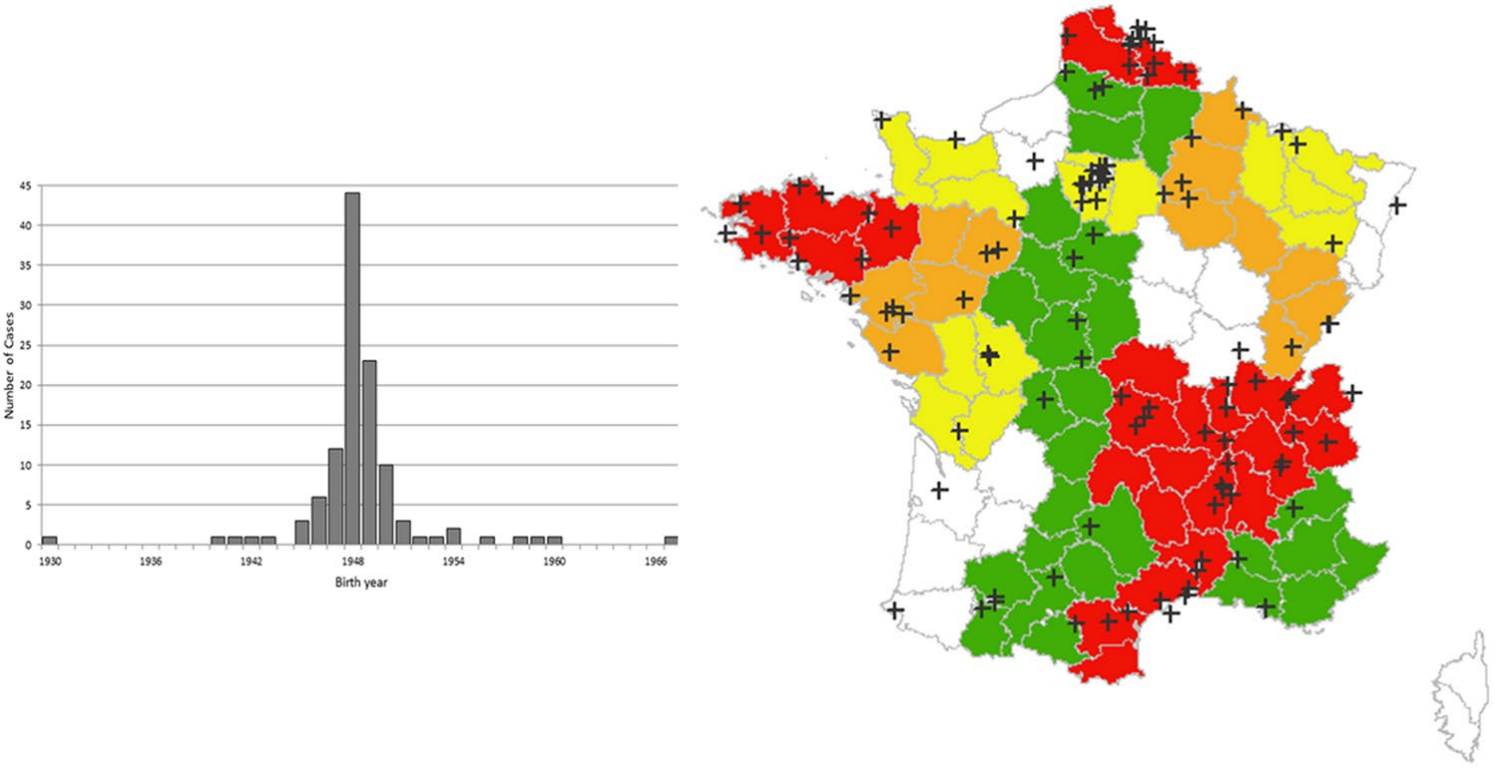
(**A**) Birth date distribution of cases by year. Cases were included from 40 to 80 years old. Note the strikingly high frequency of patients born between 1947–1950. (**B**) Geographical French repartition of EMAP patients according to their birthplace. The disease incidence is high in regions with industrialized or farming activities (in red). Disease incidences (per 10^6^ inhabitants): White no case, Green 1 to 2, Yellow 2 to 5, Orange 5 to 10, Red > 10. The French map with permission from http://jfbradu.free.fr/cartesvect/fdcfrance.htm. This map was modified with Microsoft Paint, version 6.3 (2013, Microsoft Corporation, Redmond, Washington, USA).

Full dietary and environmental data were collected for all 115 (70 women and 45 men) EMAP patients and 345 matched (by sex, age ± 5 years and place of residency) controls. AMD genetic factors were analyzed in a subset of EMAP patients and compared with a national dataset. The mean age of EMAP patients was 63.1 ± 3.6 years with 95 patients born between 1946 and 1950 (a notable peak was detected in 1948; Fig. 4).

### Dietary pattern

There was no significant difference between cases and geographically-matched controls concerning energy intake (OR = 1.04, 95%, confidence interval - CI = 0.84–1.28), protein intake (OR = 0.51 95% CI = 0.21–1.25), lipid intake (OR = 0.84 CI = 0.28–2.53), carbohydrate intake (OR = 1.00 CI = 0.61–1.63), or alcohol consumption (OR = 1.03 CI = 0.43–2.45). Regarding nutrients that have putative associations with AMD (such as lipids, macular xanthophylls (lutein/zeaxanthin), pro-vitamin A carotenoids, vitamins and minerals with antioxidant properties) no differences were detected (Table 1).

**Table 1:** Nutrition patterns in EMAP patients and controls. FA: fatty acids. The data (OR, IC, p-value) represents univariate analysis results. None of the nutrients was significant under multivariate conditional logistic model.

	Cases (n = 115)	Controls (n = 345)	OR	IC−	IC+	p
Energy (kCal/day)	2611 (±1460)	2549 (±914)	1.039	0.841	1.283	0.72
Water (g/day)	2948 (±937)	2895 (±989)	1.000	0.999	1.000	0.50
Fibers (g/day)	32.7 (±19)	34.3 (±25.7)	1.009	0.778	1.308	0.94
Proteins (g/day)	100.2 (±35.8)	106.4 (±38)	0.514	0.210	1.258	0.96
Carbohydrates (g/day)	297 (±230)	277.1 (±119)	1.006	0.617	1.639	0.98
Alcohol (g/day)	14.6 (±17)	13.8 (±15.5)	1.032	0.434	2.456	0.94
Starch (g/day)	121.0 (±65.7)	116.9 (±57.1)	1.017	0.975	1.061	0.43
Sugar (g/day)	140.8 (±165.3)	122.7 (±70.3)	1.012	0.969	1.057	0.59
Lipids (g/day)	92.9 (±52.6)	92.4 (±41.1)	0.848	0.284	2.536	0.76
FA monounsaturated (g/day)	27.6 (±15.1)	28.3 (±11.9)	0.970	0.589	1.597	0.19
FA oleic (g/day)	21.1 (±11.4)	21.7 (±9.5)	0.824	0.583	1.165	0.27
FA polyunsaturated (g/day)	12.9 (±6.2)	13.4 (±5.5)	1.370	0.447	4.200	0.32
FA saturated (g/day)	42.2 (±27.7)	40.2 (±22)	0.929	0.670	1.289	0.66
Omega 3 (g/day)	1.5 (±0.9)	1.6 (±0.9)	0.957	0.267	3.427	0.94
Omega 6 (g/day)	8.4 (±4.5)	8.7 (±4)	0.805	0.451	1.436	0.55
Cholesterol (mg/day)	301 (±138)	323 (±152)	1.426	0.822	2.474	0.04
Vitamins						
A (µg/day)	620 (±458)	786 (±673)	0.727	0.465	1.137	0.05
β-carotene (µg/day)	6.2 (±66.8)	109.8 (±839.8)	0.987	0.952	1.023	0.46
B1 (mg/day)	1.4 (±0.8)	1.4 (±0.6)	1.820	0.872	3.801	0.17
B2 (mg/day)	2.1 (±0.8)	2.2 (±0.9)	1.063	0.533	2.122	0.40
B3 (mg/day)	21.1 (±9.2)	23.2 (±11.5)	0.831	0.484	1.429	0.72
B5 (mg/day)	5.3 (±2.1)	5.7 (±2.3)	0.768	0.460	1.281	0.31
B6 (mg/day)	2.3 (±1.04)	2.4 (±0.9)	1.075	0.485	2.384	0.66
B9 (µg/day)	410 (±164)	442 (±184)	0.768	0.461	1.279	0.88
B12 (µg/day)	8.8 (±4.8)	10.3 (±6.9)	1.080	0.620	1.882	0.53
C (mg/day)	156 (±80.3)	162 (±96)	0.996	0.990	1.003	0.25
D (µg/day)	3.1 (±1.6)	3.4 (±1.7)	0.956	0.713	1.282	0.45
E (mg/day)	12.1 (±7.1)	12.2 (±5.2)	1.215	0.755	1.957	0.17
K (µg/day)	3.4 (±3.8)	3.6 (±3.6)	1.08	0.47	2.41	0.69
Micronutrients						
Calcium (mg/day)	1512 (±522)	1582 (±587)	1.000	0.998	1.001	0.71
Copper (mg/day)	3.1 (±1.4)	3.3 (±1.7)	1.349	0.795	2.288	0.26
Iron (mg/day)	20.9 (±14.4)	21.7 (±11.2)	0.818	0.487	1.374	0.08
Iodine (µg/day)	168.9 (±60.4)	179 (±69.6)	0.987	0.631	1.542	0.35
Magnesium (mg/day)	484 (±211)	497 (±195)	1.305	0.647	2.632	0.11
Manganese (mg/day)	4.6 (±1.9)	4.8 (±2.1)	0.647	0.391	1.070	0.08
Phosphorus (mg/day)	1619 (±660)	1709 (±639)	1.000	0.999	1.002	0.49
Potassium (mg/day)	3988 (±1477)	4124 (±1466)	1.000	0.999	1.001	0.46
Selenium (µg/day)	204.8 (±84.9)	202 (±88.6)	1.006	1.000	1.011	0.03
Sodium (mg/day)	3427 (±1357)	3512 (±1267)	1.000	1.000	1.001	0.89
Zinc (mg/day)	0.16 (±1.7)	0.35 (±2.2)	2.472	0.207	29.439	0.47

As dietary patterns could be variable due to regional influences in France, we calculated the Mediterranean Diet Score (MDS)^10,11^. There was no significant difference in MDS between patients (4.38, SD 1.47) and controls (4.28, SD 1.55, OR = 1.043 CI (0.908–1.199) p = 0.549). Nevertheless, there was a lower incidence of EMAP (1.96/1.10^6^) in the South-Eastern part, in comparison to the rest, of France (2.95/1.10^6^). Moreover, there were no cases reported in three departments belonging to this region (Alpes de Haute Provence, Alpes Maritimes, Var) where a Mediterranean diet and high fish consumption prevail, despite the existence of two inclusion centers (Marseille and Nice) in the vicinity.

After the onset of retinal disease, EMAP patients were more frequently supplemented with DHA/Omega and resveratrol than controls (50% for cases versus 0.3% for controls). Over the last five years, controls used more frequently vitamin D and calcium.

### Sunlight exposure

There was no significant difference between cases and controls concerning lifetime ambient solar radiation exposure. The mean exposure was 448.2 (±56.1) kJ/cm^2^/year compared to 458.9 (±72.5) kJ/cm^2^/year for cases and controls respectively (OR = 1.05 CI = 0.92–1.21). Concerning sun exposure and protection specifically during summer time, the risk of EMAP was increased for subjects not using regular protection during youth and adulthood (OR = 0.64 (0.44–0.92) and OR = 0.49 (0.33–0.73) respectively).

### Chemical exposure

As shown in Table 2, a significant association was found between professional exposures and EMAP (OR = 2.29; CI = 1.41–3.75). Indeed, 38.9% of cases used chemicals in the work place compared to 22.5% of controls. In more detail, 15% of cases used bleach compared to 4% of controls. Over 30 other chemicals were too weakly represented for a conclusion to be drawn.

**Table 2:** Sex- and age-adjusted Odds Ratios (ORs) and 95% confidence intervals (CI) between each chemical exposure variables and EMAP status (115 cases vs 345 controls).

	Cases (N = 115)	Controls (N = 345)	OR (95% CI)	P
**Personal exposure to chemicals**				
Pesticides				
No	72 (62.6%)	196 (56.8%)	1.00	Ref
Yes	43 (37.4%)	149 (43.2%)	0.43 (0.15–1.23)	0.11
Aerosols deodorants				
No	23 (20%)	56 (16.3%)	1.00	Ref
Yes	92 (80%)	288 (83.7%)	1.51 (0.83–2.74)	0.17
Anti-flea				
No	68 (59.1%)	212 (62.2%)	1.00	Ref
Yes	47 (40.9%)	124 (36.3%)	0.93 (0.58–1.49)	0.76
**Professional exposure to chemicals**				
Pesticides				
No	109 (95.6%)	323 (95%)	1.00	Ref
Yes	5 (4.4%)	17 (5%)	1.19 (0.32–4.47)	0.79
Other chemicals				
No	69 (61.1%)	262 (77.5%)	1.00	Ref
Yes	44 (38.9%)	76 (22.5%)	2.29 (1.41–3.75)	0.0008

### Birthplace and subsequent residencies

In this national clinical study, inclusion centers were chosen according to their retinal referent status and to their geographic location to allow a correct national coverage (three in the center i.e. Paris, two in the East, two in West, one in the North, and two in the South of France). The mean national EMAP incidence was 2.9/10^6^ habitants. We then calculated the incidence of EMAP disease with regards to place of birth and childhood residency (Fig. 4). This analysis disclosed an unequal distribution of disease incidence. Higher rates were found in regions with intensive farming activities, such as Auvergne (12/10^6^ habitants), Picardie (10.98/10^6^ habitants) and Brittany (8.3/10^6^ habitants). In highly industrialized areas, the incidence was also high, 26.53/10^6^ habitants in the Nord-Pas de Calais (carbon and textile industries) and 10.43/10^6^ habitants in Puy de Dôme (automobile industries).

### AMD genetic variants analysis

Genetic analysis of risk allele including those of the alternative complement pathway associated with AMD was performed in 65 EMAP patients^12–17^. Fifty-one patients were homozygous (23) or heterozygous (28) for the rs1061170 (His402Tyr) in *CFH* gene. Five and 25 patients were homozygous and heterozygous for the rs10490924 (Ala69Ser) in *ARMS2* gene, respectively. For the rs2230199 (Arg102Gly) in C3, two patients were homozygous and 20 were heterozygous. No patient had the allele rs4151667 (Leu9His) in the C2/CFB factor. Twenty-five patients were homozygous (4) or heterozygous (21) for the rs800292 (Val62Ile) in *CFH* gene. The remaining twenty-one genetic risk-factors associated with AMD (not involving the complement pathway) described by Buitendijk *et al*. were not found in EMAP patients. The score varied from −1.159 to 2.833. Forty five out of 65 EMAP patients had a score inferior to 1. Fifteen patients had a score between 1 and 2, and only 5 patients had a score between 2 and 3.

We also analyzed three other rare AMD variants that confer a risk of early onset or severe AMD. None of the 65 patients carried the CFH Arg1210Cys (rs121913059) or the C9 Pro167Ser (rs34882957) variants. Only one patient carried the Lys155Gln C3 variant (rs147859257, heterozygous state). Based on WES analysis performed in 65 EMAP patients, we excluded other retinal monogenic disorders (RetNet (https://sph.uth.edu/retnet/)), including L-ORD and late onset Stargardt disease.

## Discussion

**Figure 5:**
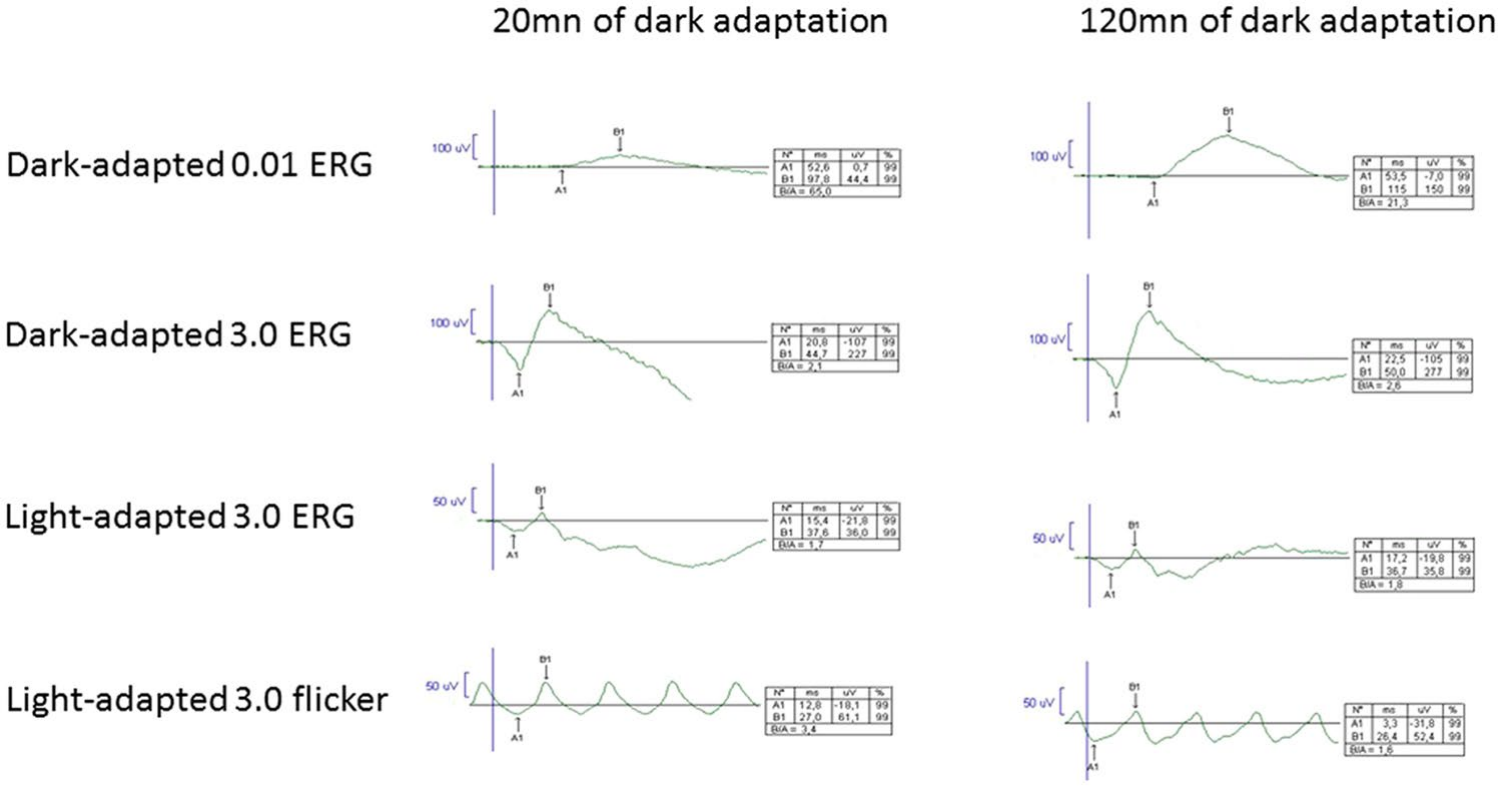
Full-field electroretinogram in EMAP patient. Dark-adapted 0.01 ERG responses are reduced after a 20 minute-dark adaptation time (ISCEV protocol), but dramatically recover after a 120 minute prolonged dark adaptation. The rod-impairment could be induced by the pseudodrusen i.e. diffuse subretinal deposits located between the retinal pigment epithelium and the inner and outer segments of the photoreceptors. Severe decrease of Light-adapted 3.0 ERG and flicker ERG responses as noted in EMAP patients, and not in AMD, suggests that cone degeneration is part of the EMAP process.

The present study highlighted a possible toxic mechanism in EMAP with a higher incidence observed in regions with industrialized or farming activities, and a toxic exposure during professional life. In AMD, fungicides may increase the risk of retinal degeneration based on the Agricultural Health Study of farm families from Iowa and North Carolina (1993–1997)^18,19^. The role of long-term/low-dose exposure to pesticides (organophosphates, paraquat) is well documented in Parkinson disease, which is the consequence of a degeneration preferentially affecting the dopamine-synthesizing neurons of the nigrostriatal neuronal pathway^20–23^. Even if the molecular mechanisms of the neuronal degeneration are not completely identified, most pesticides produce oxidative or endoplasmic reticulum stress, mitochondrial alterations, and finally neuronal cell loss. EMAP may share a toxic predominant neuronal degeneration with Parkinson disease. The EMAP-associated neuronal degeneration could involve rods, cones or both. Despite a systematic inaugural night blindness, electroretinogram (ERG) studies did not suggest a major rod cell death but a rod dysfunction. Rod responses are notably reduced after a 20 minute-dark adaptation time (ISCEV protocol), but dramatically recover after a 120 minute prolonged dark adaptation (Fig. 5). This rod-impairment could be induced by the pseudodrusen i.e. diffuse subretinal deposits located between the retinal pigment epithelium and the inner and outer segments of the photoreceptors. Systematic and severe decrease of photopic single flash and flicker ERG responses as noted in EMAP patients (Fig. 5), and not in AMD, suggests that cone degeneration is part of the EMAP process. In human and monkey retinas, cone density increased from a 7 mm (35°) of eccentricity line (20 000 cones/mm2) to the fovea peak cone (150 000 cones/mm2). This cone distribution correlates with the pattern and extent of EMAP macular atrophy and 25–35° central scotoma. In addition, EMAP atrophic paving stone lesions developed in the far periphery of the retina, which is a cone-enriched rim deprived of rods that begins 13 mm from the fovea and peaks at 1 mm from the ora serrata^24,25^. Thus, EMAP could be distinct from AMD and revisited as a toxic neurodegenerative cone disease with early, severe and rapid cone apoptosis. An additional deleterious effect of sunlight exposure could also be considered as patients reported a less frequent use of sun glasses. EMAP patients did not show clinical signs of any other neurodegenerative disorders and did not report any family history of such disorders in the questionnaire.

Mediterranean diet and consumption of virgin olive oil reduce the incidence of chronic or neuro-degenerative diseases. Virgin olive oil phenolics have positive effects on antioxidant status, antimicrobial activity and on inflammation as they are natural nonsteroid anti-inflammatory drugs. It is noteworthy that EMAP incidence is particularly low in the southern Mediterranean region (Provence, Côte d’Azur) suggesting a protective role of this diet. Such a protective impact is also reported in AMD on its progression towards the neovascular and atrophic forms^11,26,27^. However, no such difference for Mediterranean diet was detected at the individual level in our study probably because EMAP patients were geographically (i. e. with a similar diet) matched with controls.

As a combined abnormal inflammatory response with abnormal CH50 and C3 serum levels, without ocular inflammation or neoplastic diseases were documented in the first study, here we focused on risk alleles for AMD that involve different complement factors of the alternative complement pathway. There was no significant difference for AMD variants including rs1061170 (His402Tyr) and rs800292 (Val62Ile) in *CFH*, rs10490924 (Ala69Ser) in *ARMS2*. The variant rs2230199 (Arg102Gly) in C3 was significantly most frequent in EMAP patients (Table 3). Abnormal alternative complement pathway response could be also involved in EMAP disease that shares with AMD pseudodrusen and not classic AMD drusen. These common AMD single nucleotide variants are also associated with increased prevalence of PSD^28–31^. In our cohort of 65 patients, we did not find any association with rare AMD variants, in particular Arg1210Cys in CFH that confers a risk of a 6-year earlier onset of AMD and of a subtype of extensive large drusen throughout retinal vascular arcades. With an allelic frequency of 0.005922% in ExAc (European non-Finnish population), one patient carried the rare variant C3 Lys155Gln (rs147859257) that results in resistance to C3 protein regulation and therefore alternative complement pathway amplification^14^. Saksens and colleagues reported that patients with advanced atrophic AMD carried these rare variants more frequently than patients with neovascular AMD (11.8% vs 4.8%)^13^. These rare AMD variants may not be linked to EMAP or may have been underestimated in this study due to the number of EMAP patients.

**Table 3:** Analysis of the five alleles of the alternative complement pathway associated with AMD noted in EMAP patients (n = 65) versus ExAc data non-Finnish European population). WT: Wild type.

	EMAP	ExAc	OR (95% CI) and p-value	
			WT vs mutation	heterozygous vs homozygous
CFH				
rs1061170	N = 65	N = 33026		
Wild type	14	4764	1.63 (0.9; 2.95) p = 0.103	
Heterozygous	28	15777		0.95 (0.55; 1.67) p = 0.894
Homozygous	23	12485		
CFH				
rs800292	N = 65	N = 33208		
Wild type	40	19214	1.17 (0.71; 1.93) p = 0.548	
Heterozygous	21	12110		0.82 (0.28; 2.39) p = 0.710
Homozygous	4	1884		
ARMS2				
rs10490924	N = 65	N = 33304		
Wild type	35	20113	0.77 (0.47; 1.25) p = 0.281	
Heterozygous	25	11601		0.69 (0.26; 1.81) p = 0.438
Homozygous	5	1590		
C3				
rs2230199	N = 65	N = 33353		
Wild type	43	26499	0.51 (0.3; 0.85) p = 0.008	
Heterozygous	20	5435		2.61 (0.61; 11.18) p = 0.179
Homozygous	2	1419		

To conclude, EMAP could be revisited as a neurodegenerative disorder with predominant cone apoptosis. This study provides insights that EMAP could be caused by lifelong toxic exposure combined with a chronic inflammation and abnormal complement pathway regulation. This leads to diffuse subretinal deposits and cone apoptosis around the age of 50 with characteristic extensive macular atrophy and paving stones in the far peripheral retina. Additional WES analysis is now warranted in order to identify other genetic risks factors of this early onset macular atrophy with diffuse pseudodrusen.

## Subjects and Methods

### Study design

This national research program was validated and financed by the French Health Ministry and was conducted between May 2011 and July 2014. Ten referent national centers specialized in retinal diseases were mandated to recruit 115 patients with the help of all other public and private ophthalmologists. Four clinical research centers (Montpellier for the South of France, Lille for the North, Tours for the West and Dijon for the East) included three controls for each EMAP case, matched by actual residency area, age (±5 years) and sex. Controls were solicited from volunteer lists.

This research followed the tenets of the Declaration of Helsinki. The design of this study was approved by the local Ethical Committee (CPP Sud Méditerranée IV, decision March 8^th^, 2011). All methods were performed in accordance with the relevant guidelines and regulations. Informed consent has been obtained for each participant of this study.

### Population

Common inclusion criteria for cases and controls were (1) women and men aged 40 to 80, (2) Caucasian origin.

### Cases

Inclusion criteria for the EMAP patients were (1) onset of functional signs before the age of 55 years (2) macular patch of atrophy with a large vertical axis (3) diffuse peripheral pseudodrusen. Color fundus images were performed with Topcon Imagenet (Ophthalmic Imaging Systems, Japan) or Nidek non-mydriatic automated fundus camera AFC 330 (Nidek Inc, Japan). Autofluorescence imaging and SD-OCT imaging were performed with Combined Heidelberg Retina Angiograph + OCT Spectralis device (Heidelberg Engineering, Dossenheim, Germany). When the two national coordinators (IM and CH) disagreed on clinical features, the patient was not included. Full-field electroretinography (ERG) was performed according to the guidelines of the International Society for Electrophysiology of Vision (ISCEV) using a Ganzfeld apparatus (Ophthalmologic Monitor Metrovision, Pérenchies, France)^32^.

### Controls

Color fundus images were systematically performed with a non-mydriatic device (Nidek non-mydriatic automated fundus camera AFC 330, Nidek Inc, Japan) in order to exclude subjects with any retinal disease or atrophic macular lesions. Controls older than 70 years with uncomplicated drusen could be included.

### Dietary patterns

Nutritional data were collected using a validated food frequency questionnaire (FFQ) that recorded the usual food intakes^33–35^. The FFQ included 250 items and portions were estimated using a validated set of photographs (ref.^19^) arranged by food type and meal pattern. In the analysis, the intakes were expressed in daily consumption and seasonality of some products was taken into account. The food composition was determined with Ciqual 2013 table (National Safety Agency for food, environment and activities - ANSES, https://www.anses.fr).

### Sunlight exposure

All participants completed a self-administered questionnaire relative to sun exposure and protection during summer time for 3 periods of their life: youth (<20 year-old), adulthood and currently (last five years). For each period of life, a first item recorded the daily duration of exposure to sunlight (“less than 2 hours”, “2 to 5 hours”, “more than 5 hours”). A second item recorded the frequency (“never” “rarely” “often”) of protection against the sun (hat or sunglasses). In addition, we carefully noted all the residency locations and their respective durations over the life time of the individual. For each participant, the average of ambient solar radiation (ASR) was calculated using data from Météo France (www.meteofrance.com/climat), taking into account the latitude and the duration of each residency locations, together with the duration of daily sunshine exposure^36^.

### Chemical exposures

Profession of each patient and its associated toxic risk were identified. In addition, all subjects completed a 25-item chemical exposure questionnaire relative to chemicals used in professional and in non-professional activities. Chemical exposures were recorded across “Yes/No” questions and frequency levels from “once a day” to “few times a year”. In addition, participants had to indicate the names of the products used (brand, type).

### Environmental factors and Geographic incidence

All EMAP patients gave information on their birthplace and their subsequent residencies until inclusion to assess the lifelong environmental exposure (farming and industrialized areas). To avoid any bias associated with recruitment centers, we evaluated the incidence of EMAP by place of birth and childhood residency, and normalized to the number of inhabitants reported in 1950 (corresponding to the birth peak).

### Statistical analysis

The control versus EMAP cohorts were the same as reported in Douillard *et al*.^9^. For each potential risk factor, age-, and gender-adjusted odds-ratios (OR) for EMAP were calculated using conditional logistic regression, with EMAP as the dependent variable. In a second step, a multivariate conditional logistic model was performed with age, gender and all independent variables that were close to significance in the first model (p < 0.20). Non-significant variables (p < 0.10) were then deleted from the model following a backward stepwise procedure to obtain the final multivariate model. For genetic analysis, χ² test or exact Fisher test were used appropriately depending on the distribution.

### Genetic analysis

Whole exome sequencing (WES) was performed in 65 EMAP patients. These data were compared with non Finnish European population of ExAC database. We studied common (rs1061170, His402Tyr in *CFH* gene, rs800292, Val62Ile in *CFH* gene, rs10490924, Ala69Ser in *ARMS2* gene, rs2230199, Arg102Gly in C3, rs4151667, Leu9His in the C2/CFB factor) and rare (rs121913059, Arg1210Cys in CFH - rs34882957, Pro167Ser in C9 - rs147859257, Lys155Gln in C3) variants. We also estimated the genetic risk score based on a maximal gene-environmental model including age, sex, environmental and ocular factors, and 26 genetic AMD risk variants^37^. Raw sequence alignment and variant calling were carried out using Illumina CASAVA 1.8 software. CASAVA performs the alignment of reads to the human reference genome (hg19) using the alignment algorithm ELANDv2, and then calls single nucleotide variants (SNVs) and short insertions and deletions (indels) based on allele calls and read depth. We used VEP version 83 (http://www.ensembl.org/info/docs/tools/vep/index.html) and an Integragen in-house pipeline to annotate each variant according to its presence in the 1000 Genome, ExAC or Integragen database, and according to its functional category (synonymous, missense, nonsense, splice variant, frameshift or in-frame indels. Some AMD risk alleles discovered in GWAS studies are located outside of the coding regions captured by WES and would therefore not be assessed by this method.
